# PlanMine – a mineable resource of planarian biology and biodiversity

**DOI:** 10.1093/nar/gkv1148

**Published:** 2015-11-17

**Authors:** Holger Brandl, HongKee Moon, Miquel Vila-Farré, Shang-Yun Liu, Ian Henry, Jochen C. Rink

**Affiliations:** Max Planck Institute of Molecular Cell Biology and Genetics, Pfotenhauerstrasse 108, 01307 Dresden, Germany

## Abstract

Planarian flatworms are in the midst of a renaissance as a model system for regeneration and stem cells. Besides two well-studied model species, hundreds of species exist worldwide that present a fascinating diversity of regenerative abilities, tissue turnover rates, reproductive strategies and other life history traits. PlanMine (http://planmine.mpi-cbg.de/) aims to accomplish two primary missions: First, to provide an easily accessible platform for sharing, comparing and value-added mining of planarian sequence data. Second, to catalyze the comparative analysis of the phenotypic diversity amongst planarian species. Currently, PlanMine houses transcriptomes independently assembled by our lab and community contributors. Detailed assembly/annotation statistics, a custom-developed BLAST viewer and easy export options enable comparisons at the contig and assembly level. Consistent annotation of all transcriptomes by an automated pipeline, the integration of published gene expression information and inter-relational query tools provide opportunities for mining planarian gene sequences and functions. For inter-species comparisons, we include transcriptomes of, so far, six planarian species, along with images, expert-curated information on their biology and pre-calculated cross-species sequence homologies. PlanMine is based on the popular InterMine system in order to make the rich biology of planarians accessible to the general life sciences research community.

## INTRODUCTION

Planarians, a large group of worms with generally flattened body architecture, are best known for their regenerative abilities (1–3). The worms have the astonishing capability to regenerate complete and perfectly proportioned animals from tiny tissue pieces. Planarians are also of key interest to stem cell research, owing to their abundant adult pluripotent stem cells that continuously renew all organismal cell types (4–6). Further intriguing features include *de novo* germ line regeneration (7), reproductive strategy dependent ageing phenomena (8) and food-supply dependent growth/degrowth (9). So far, the planarian research community studies mostly two model species *Dugesia japonica* and *Schmidtea mediterranea* (Smed). However, hundreds of planarian species exist worldwide. Some are known to be regeneration impaired or even entirely regeneration-deficient (10–13), others differ in tissue turn-over kinetics (14) and life spans range from seemingly unlimited in asexual strains to a few months in species with a seasonal life history (15–17). Planarians are also cheap and easy to maintain in the laboratory. Our lab and others are currently establishing systematic live collections of ‘wild’ planarian species in order to make their rich phenotypic diversity accessible to comparative analysis.

Much of recent planarian research, especially the work with ‘wild’ species, involved next generation sequencing (NGS) techniques. For animals brought into the lab literally out of the wild, transcriptomes and transcriptome comparisons with characterized species provide powerful entry points into the analysis of molecular mechanisms. For model species, transcriptomes and RNA sequencing (RNA-Seq) experiments enable querying of gene expression dynamics and the design of *in situ* or RNA interference (RNAi) probes for querying expression patterns or gene functions, respectively. However, the enthusiastic embrace of NGS technologies has also brought up new challenges in the planarian research community and beyond, including issues of reproducibility and standards in the face of multiple independently assembled transcriptomes.

The *S. mediterranea* genome database (SmedGD) (18,19) already provides Smed transcriptome and genome data generated by the SmedGD host lab. PlanMine (http://planmine.mpi-cbg.de/) aims to provide an easily accessible and minable repository of general planarian sequence data. Our dual mission objective is to first, provide opportunities for comparing and mining planarian transcriptomes and RNA-Seq data sets created across the community; second, to catalyze the comparative analysis of the phenotypic diversity amongst planarian species. We chose the popular InterMine data warehouse system (20) for PlanMine in order to facilitate comparisons with other model systems and to make the fascinating biology of planarians accessible to the general life sciences research community.

## PLANMINE OVERVIEW

At its core, PlanMine is a minable repository of richly annotated transcriptomes of planarian species.

Currently there are two sources for transcriptomes deposited in PlanMine. Firstly, transcriptomes assembled using a pipeline established by the Rink lab (see the online help manual of PlanMine for details, http://planmine.mpi-cbg.de/planmine/PlanMine_Help.html#assembly). Secondly, multiple Smed transcriptomes contributed by the community and assembled by different strategies (21–27). Contributed transcriptome assemblies are left untouched at a sequence level but are included in our subsequent transcriptome annotation pipeline to ensure data consistency. The ‘Data Sources’ tab on the home page provides an overview of transcriptomes and contributors. For in-house assembled transcriptomes, we additionally provide a detailed assembly report with multiple quality control parameters, which are explained in detail in the online help manual of PlanMine (http://planmine.mpi-cbg.de/planmine/PlanMine_Help.html#assembly-reports).

**Figure 1.**
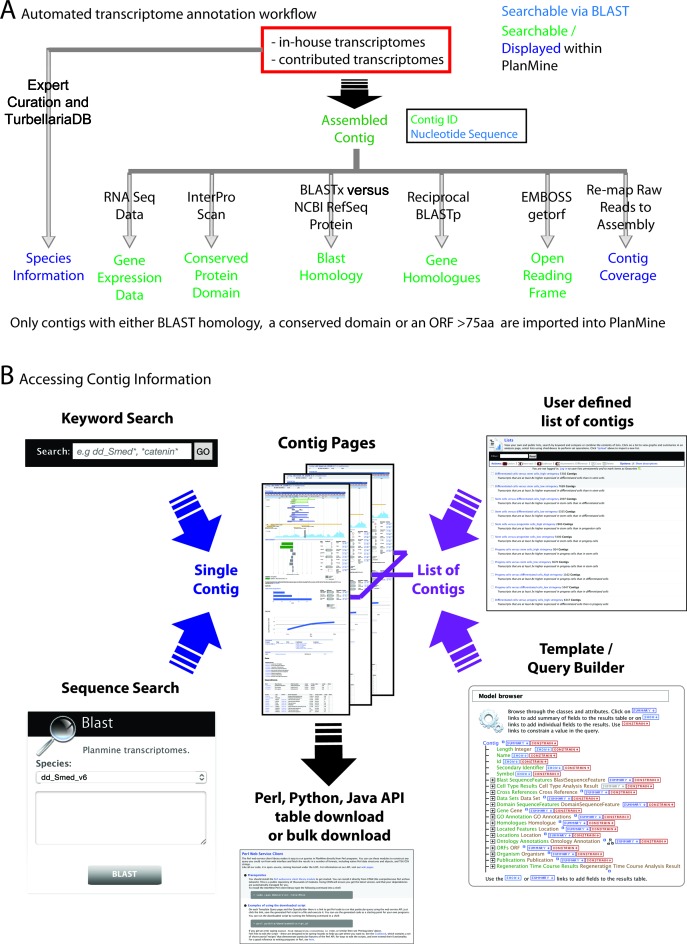
(**A**) Annotation steps performed on all assemblies in PlanMine. Text color coding indicates the accessibility of a particular annotation type. (**B**) Scheme of data mining and export options in PlanMine. See text for details.

As a prerequisite for meaningful comparisons, all transcriptomes in PlanMine are annotated using an automated pipeline (Figure 1A). Briefly, our pipeline annotates contigs by BLAST homology to sequences in the NCBI RefSeq protein database (28) using BLASTX (29), protein domains using the InterProScan suite (30) and open reading frames (ORF) using the EMBOSS getorf tool (31). Additionally, likely orthologous contigs in other PlanMine transcriptomes are annotated via reciprocal BLASTP using the longest ORF for each ‘gene’ (set of contigs belonging to one trinity graph component (32); see the online help manual of PlanMine for details on sequence identifiers http://planmine.mpi-cbg.de/planmine/PlanMine_Help.html#contig-identifier-naming-scheme). We annotate gene ontology (GO) terms (33) based on GO terms associated with homologous proteins and, for in-house assemblies, we align the raw read data onto the final assembly to create read coverage tracks for each assembled contig. We further use the annotations to filter assemblies: Only contigs that have an ORF longer than 75 amino acids, an annotated domain or have significant BLAST homology are incorporated into PlanMine. Supplementary Material S1 summarizes the parameter settings and reference information at the time of publication. The corresponding section of the PlanMine help manual (http://planmine.mpi-cbg.de/planmine/PlanMine_Help.html#reference-information) will always provide an up-to-date reference. Further layers of annotation, described in more detail below, include differential transcript expression in published RNA-Seq data sets and expert-curated information on the planarian species that are represented by a transcriptome in PlanMine.

The InterMine framework provides multiple ways to query/analyze the data held within PlanMine and also easy export options for the retrieval of results and sequence information (Figure 1B). Firstly, the keyword search box in the upper right hand corner of the home page allows searches for specific contig IDs or specific annotations (e.g. domain names, BLAST homology, etc). Searches generally result in lists of contig IDs associated with the search term. Clicking a contig ID brings up the respective contig page showing all available annotation information for this specific sequence. A second important search modality is sequence homology searches via BLAST. We integrated the SequenceServer software (http://sequenceserver.com), which can be accessed either by using the BLAST search box on the homepage or the ‘BLAST’ tab at the top of the home page. Again, the BLAST result pages link directly to individual contig pages.

A powerful feature of the InterMine framework is that it not only allows one at a time search modes, but also the analysis of lists of contigs. Described in more detail below, these features allow such operations as retrieving all contigs with a particular domain annotation from a particular planarian species, retrieving all differentially expressed genes from a specific RNAi experiment or performing GO-term enrichment analysis on a list of contigs. Export options include Excel tables (e.g. of enriched GO terms and associated contigs), FASTA files of sequence information, or the option to download all transcriptomes in PlanMine, either using the list export option or the download option under the ‘Data Sources’ tab on the home page. It is also possible to access PlanMine via the InterMine provided API, thereby enabling the programmatic use of PlanMine in custom data analysis workflows.

Note that PlanMine so far does not assign or use gene names (see the online help manual for background on the PlanMine contig naming scheme, http://planmine.mpi-cbg.de/planmine/PlanMine_Help.html#contig-identifier-naming-scheme). However, we incorporated a list of published gene sequences that will be regularly updated. Searches for published gene names (please use wildcards to avoid false negatives, e.g. *catenin-1*) consequently bring up sequences of published genes, which can then be associated with their corresponding PlanMine transcripts via BLAST or the pre-calculated orthologue listings in other Smed assemblies on the contig page of published gene (see below).

Overall, these features provide an easily accessible repository of planarian transcriptome information and one of the few opportunities so far for systematic gene sequence analyses within the superphylum Lophotrochozoa.

## COMPARING SMED ASSEMBLIES

**Figure 2.**
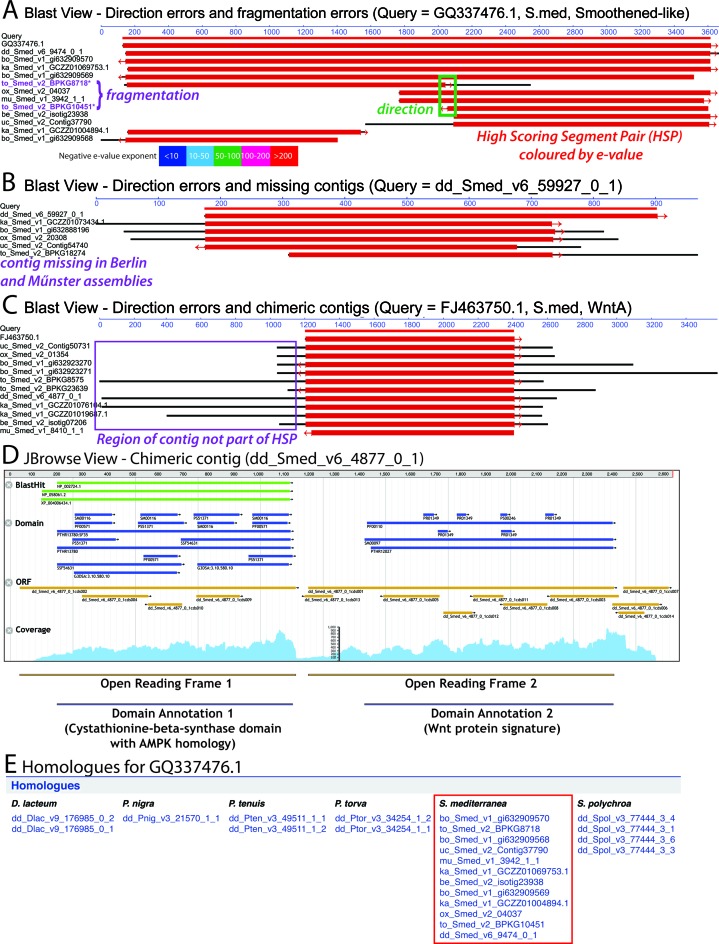
(**A**–**C**) Contig comparison between different *Schmidtea mediterranea* (Smed) assemblies, using the customized BLAST viewer. BLAST high scoring segment pairs (HSPs) are color coded based on e-value (red to blue), regions of subject contigs outside of an HSP are shown in black and contig direction is indicated by an arrow at the appropriate end of an HSP. Purple and green text denotes viewer features designating specific assembly mistakes. Using a contig sequence from any assembly as query and searching against all other PlanMine Smed assemblies allows the identification of the following assembly error categories: (**A**) Contig fragmentation and directionality errors. Fragmentation is evident by two independent contigs in the same assembly (here the Toronto assembly), both producing high scoring HSPs to complementary regions of the query. Contig directionality can be inferred from the directionality arrows. (**B**) Contigs missed in a particular assembly, evident here by the absence of an HSP in the Munster or Berlin transcriptomes. (**C**) Possible chimeric fusions with unrelated transcripts, indicated here by the unusually long HSPs of contigs in the Toronto and Dresden assemblies. (**D**) Annotated screenshot of the JBrowse viewer embedded in every contig page, here of one of the chimeras shown in C. The JBrowse view provides additional information in the form of BLAST homology to RefSeq proteins, domain annotations, open reading frames and transcript read coverage. The existence of two long open reading frames in the contig, their specific domain annotations and the characteristic 3′ drop in contig coverage between the two ORFs all provide additional confirmation for the chimeric nature of the contig. (**E**) Example of the pre-calculated homology information provided on each contig page. Homologues listed under *S. mediterranea* (framed in red) are likely corresponding contigs in different assemblies.

The first objective of PlanMine is to provide a platform for comparisons amongst the multiple Smed transcriptomes that are currently in use within the planarian research community. No single *de novo* assembled transcriptome is perfect, making the cross-validation of contigs between independent assemblies an important concern. The integration of the SequenceServer tool (http://sequenceserver.com) allows facile BLAST searches against all Smed assemblies. We have further custom-developed a result viewer that aids the at-a-glance detection of multiple common assembly mistakes (Figure 2A–C). These include fragmented or miss-oriented contigs (Figure 2A), contigs missed in specific assemblies (Figure 2B) or likely chimeras between unrelated transcripts (Figure 2C). As shown in Figure 2D, the JBrowse viewer (34) embedded in every contig page can provide additional evidence for chimerism (e.g. multiple reading frames with unrelated BLAST homologies or domain annotations), as well as identifying small insertions/deletions in contigs resulting in open reading frame disruption. The pre-calculated orthologous transcripts in other Smed assemblies at the bottom of every contig page (Figure 2E) and the ‘Compare Smed Assemblies’ tab on the homepage provide further useful shortcuts for comparing and retrieving orthologous contigs from different Smed assemblies.

PlanMine also provides tools for objective comparisons at the whole transcriptome level. The list of published Smed transcripts (accessible either via the ‘Data sources’ and ‘Transcriptomes’ tabs or by FASTA export of the respective list from the ‘List’ tab) can be used as a gold standard for assessing and comparing the degree of coverage of present and future assemblies. Generally, we envisage the future integration of Smed genome information as crucial milestone toward a community standard transcriptome and we have explicitly designed the PlanMine data structure with this goal in mind.

PlanMine therefore allows the identification of the likely ‘best’ transcript amongst multiple independently assembled and imperfect transcriptomes, as well as opportunities for objective comparisons among existing assemblies.

## INFERRING PLANARIAN GENE FUNCTIONS

**Figure 3.**
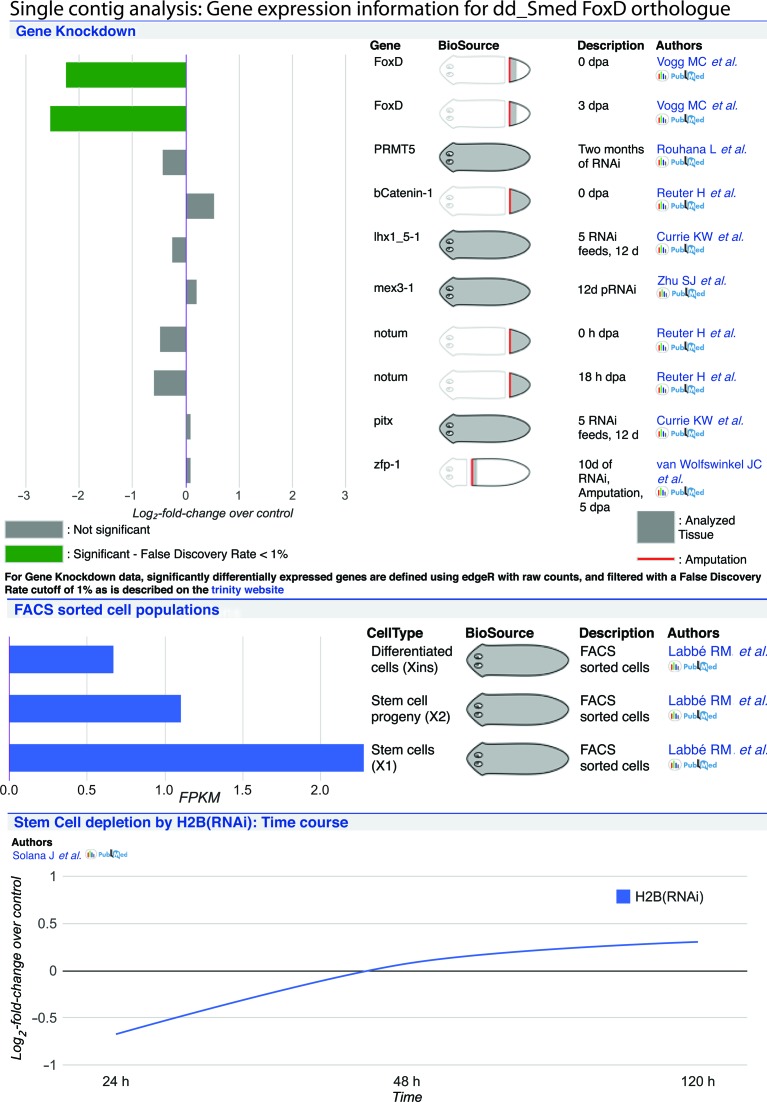
Screenshot of the gene expression information section of a contig page. Differential expression (DE) data are subdivided into three categories. Top: Gene knock-down experiments, with the bar graph representing the log2 fold-change relative to the control data set and bar color coding whether or not the change is significant by Trinity DE pipeline default settings. Centre: Tissue/Cell type specific sequencing, with the bar graph representing the FPKM of the transcript in the data set. Bottom: Time course data, in this case an H2B RNAi time course tracing contig expression levels as log2 fold change relative to the provided zero time point control. In general, the gene/cell type, BioSource and Description columns to the right of the data visualization provide information on specific experimental conditions, e.g. the name of the gene targeted by RNAi in the gene knock-down category. The standardized cartoons provide a visual summary of the type of tissue and regeneration paradigm used. The icons at the right link to the publication originally reporting the data set and to the raw data.

A second objective of PlanMine is to provide insights into potential functions of planarian genes. A dedicated page for each individual contig serves as central hub, summarizing all the available data. The domain and BLAST homology annotations in the embedded contig viewer window (Figure 2D) provide first functionally relevant annotations. To reduce the propagation of BLAST homology annotation mistakes, we report three BLAST homologues of preferred model organism homologues (human, mouse and *Drosophila*). Homologues in other species are only reported when these preferred organisms do not have matches that meet our quality criteria (see the online PlanMine help manual for details, http://planmine.mpi-cbg.de/planmine/PlanMine_Help.html#blast-annotation). We also assign GO terms on basis of BLAST homology (see the online PlanMine help manual for details, http://planmine.mpi-cbg.de/planmine/PlanMine_Help.html#gene-ontology-information). The GO terms associated with a contig are reported on the contig page and can be mined via PlanMine (see below). A further important source of potential gene function information is the integration of published RNA-Seq experiments. The gene expression graphics embedded in the contig page (Figure 3) provide at-a-glance summaries of the contig's expression dynamics under a diverse range of experimental conditions, so far including various gene knock-downs (top) (25,35–39), expression levels in stem cells, progenitors and differentiated cells (centre) (24) as well as an RNAi time course aimed at identifying stem cell genes (bottom) (40). It is important to stress that the fold-change and significance of the Trinity differential expression (DE) analysis pipeline (32) that we use for re-mapping the published data may differ from those reported in the original publications. For this reason, we also provide link-outs to the original publication of each data set and the respective raw data files. We further provide lists of contigs enriched in stem cells, progenitors or differentiated cells (derived from the above data), which are accessible via the ‘Lists’ tab and should be useful for more general explorations, within PlanMine, of the planarian stem cell compartment. Mapping and PlanMine integration of new data sets have been set up as automated workflows, enabling the rapid incorporation of new data sets. We therefore specifically encourage the community to submit newly published RNA-Seq data sets to PlanMine. Further details concerning the submission process are available in the help manual (http://planmine.mpi-cbg.de/planmine/PlanMine_Help.html#submit-data-to-planmine).

Overall, the expression profile data in conjunction with the various annotation layers provide a powerful basis for generating testable hypotheses or exploring possible conservation of gene functions between planarians and other organisms.

## MULTI-CONTIG DATA MINING

**Figure 4.**
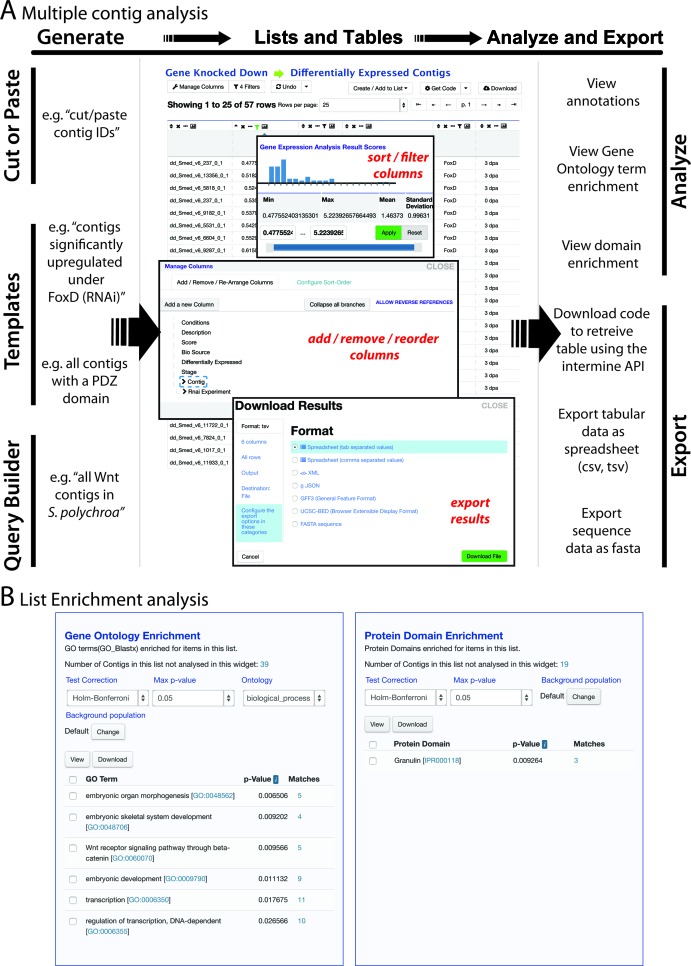
(**A**) Scheme of multi-contig analysis options within PlanMine, see text for details. (**B**) Screenshot of the customized GO term and Domain enrichment widget performed on a list of genes significantly upregulated upon FoxD knock-down.

A third objective of PlanMine is to enable the extraction and analysis of sets of genes (Figure 4). This includes operations such as performing GO term enrichment analysis on differentially expressed contigs or user-defined lists, the retrieval of all members of a particular gene family in a given planarian species or comparisons between different lists (Figure 4A). Lists of contig IDs are central to these operations and can be generated by a variety of ways. Using the ‘create’ sub-heading of the ‘Lists’ tab on the homepage, lists of contig IDs can be simply pasted into the provided window, e.g. by copy/pasting a column out of an Excel file. This method provides an easy way of uploading your own data sets for PlanMine analysis. Saving and retrieval of private data require a password protected user account, which can be set up via the ‘Log in’ tab on the home page. A second way of generating lists is by saving the output of queries. We provide a number of useful queries on the home page and under the ‘Templates’ tab, including extraction of all contigs with a given domain annotation in a given transcriptome or all genes that are significantly up or down-regulated in one of the integrated gene knock-down experiments. The InterMine QueryBuilder (20) is the tool behind such multi-relational searches. A short tutorial in the online help manual of PlanMine illustrates the use of this powerful tool (http://planmine.mpi-cbg.de/planmine/PlanMine_Help.html#tutorials). Further, we provide a number of predefined lists via the ‘view’ sub-heading of the ‘Lists’ tab on the homepage, including the stem/progenitor/differentiated cell lists mentioned above or entire transcriptomes for whole transcriptome analyses.

Once a list of contig IDs has been generated, the data are displayed as a table. The ‘Manage Columns’ tab (tool icon, top left corner of the table view page) allows the association of the various annotation layers with the listed contig IDs, e.g. the addition of a BLAST homology column to a list of contig IDs (note that per InterMine default, contigs without an annotation in the columns are no longer displayed). The four action buttons above each column allow sorting the table by columns, column removal, column hiding or displaying the data distribution within this column. The option of defining specific filters offers further options for restricting the displayed data to the contigs of interest. The ‘Download’ button offers a variety of data export options, for example as an Excel table or FASTA file. We further utilize an enrichment analysis widget (Figure 4B) that automatically performs basic GO term and protein domain enrichment analysis amongst the listed contigs. Note that the default background is always assumed to be the full transcriptome from which the contig IDs are derived, but any saved list of contig IDs can be manually specified as background. Another useful feature is the ability to merge, subtract and extract the union between different saved lists via the action icons in the header line of the ‘view’ lists page, allowing for example the extraction of genes that are both differentially expressed under an RNAi condition and also enriched in stem cells.

Collectively, these features provide a wide range of useful data mining operations, the depth and scope of which we expect to increase rapidly with the integration of new data sets and types.

## INTER-SPECIES COMPARISONS

**Figure 5.**
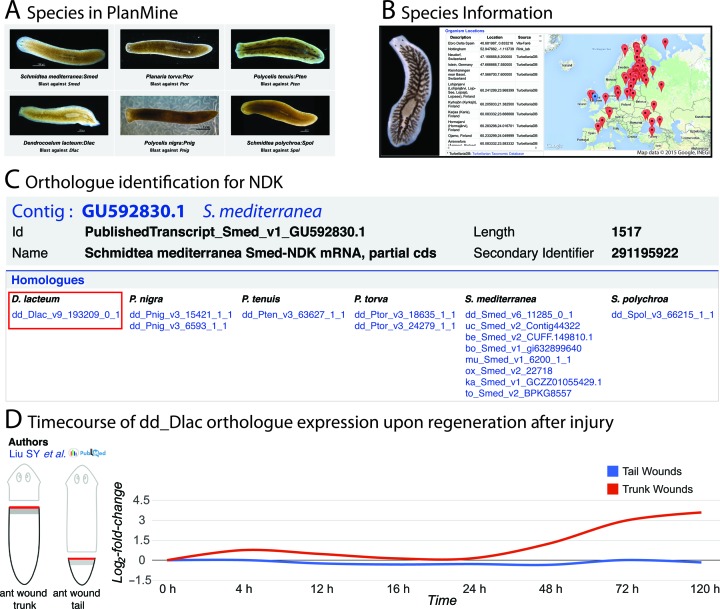
Exploring and comparing different planarian species in PlanMine. (**A**) Home page picture icons of the species currently represented in PlanMine. (**B**) Screen shots of items on the *Dendrocoelum lacteum* species page. The image displays a recently fed animal with the ingested food filling the gut branches. The red icons on the map display the coordinates of sampling locations of the species courtesy of the Turbellarian database; the blue icon designates the sampling location of the specimens that were sequenced for the PlanMine transcriptome. (**C**) Workflow for identifying the Dlac orthologue of NDK. A text search for *NDK* brings up the published contig of Smed-NDK (top). The contig page of the published transcript lists the ID of the orthologous contig in *Dendrocoelum lacteum*, dd_Dlac_v9_193209_0_1 (middle; framed in red). Clicking the dd_Dlac_v9_193209_0_1 link highlighted opens the contig page of Dlac-NDK including (**D**) a timecourse of expression upon regeneration after injury.

A fourth objective of PlanMine is to enable sequence comparisons between different planarian species. The picture icons on the home page designate the species currently in PlanMine (Figure 5A). Please note the four letter acronym of the species names that are used as prefix in contig names, e.g. *Dendrocoelum lacteum* = Dlac. Clicking the picture icons brings up the species pages, which provides expert curated information on distribution, life history and interesting phenotypes of the species, as well as high resolution pictures aiding in species identification (Figure 5B). The link-out to the Turbellarian database (http://turbellaria.umaine.edu/) integrates taxonomic information.

The non-Smed transcriptomes were assembled with the Rink lab transcriptome assembly pipeline (see the online help manual of PlanMine for details, http://planmine.mpi-cbg.de/planmine/PlanMine_Help.html#assembly) and as for Smed transcriptomes, we provide an overview of assembly statistics and detailed assembly reports under the ‘Data Sources’ tab of the home page. Transcriptomes can be searched separately or all at once, using the BLAST link on the species pages or via the home page. The use of the check boxes permits BLAST searches against single- or multiple planarian species in PlanMine. The inter-relational data architecture of PlanMine described above is ideal for inter-species comparisons, allowing for example the restriction of searches to a specific transcriptome, e.g. ‘all Wnt genes in Spol’. Further, we provide pre-calculated sets of homologous transcripts also on the species level. ‘Homologues’ are identified by a reciprocal blastp (e-value < 0.001) analysis between the longest ORFs of each trinity graph component, thus actually representing likely orthologous contigs. Figure 5C illustrates the use of these data for identifying the Dlac homologue of a Smed gene. Dlac is currently the only ‘new’ species in PlanMine for which RNA-Seq experiments have been published, specifically a time course comparison between head regenerating wounds in the anterior body half and non-head regenerating wounds in the posterior body half (10). The availability of these data in PlanMine (Figure 5D) permits mining operations aimed at identifying Dlac head specification genes and, in conjunction with the expression dynamics of orthologous Smed contigs, possibly general planarian head specification genes.

Overall, PlanMine allows inter-species comparisons at the sequence level. As more RNA-Seq data on other ‘wild’ species becomes available, the inter-relational data architecture of PlanMine will offer increasingly powerful comparative analyses of the rich phenotypic diversity amongst different planarian species.

## FUTURE PLANS

PlanMine is meant as a long-term resource for the planarian research community and anyone interested in planarians. Back-up procedures are in place to provide URL-access to older versions. Largely automated data import routines and dedicated support staff enable the integration of new RNA-Seq experiments or transcriptomes as they become publically available. The data architecture of the InterMine system further permits the expansion of the types of data stored in PlanMine. The integration of genome information is planned for the near future and we further envisage the integration of community-wide collaborations, such as systematic catalogs of gene expression patterns or RNAi screening results. Further, the planarian species collections that our lab and others are establishing are rapidly increasing the range of species available for investigation. As information storage and mining hub, PlanMine aims to catalyze the comparative analysis of the rich phenotypic diversity that planarians offer.
